# Diffusiophoretic Particle Penetration into Bacterial Biofilms

**DOI:** 10.1021/acsami.3c03190

**Published:** 2023-07-03

**Authors:** Ambika Somasundar, Boyang Qin, Suin Shim, Bonnie L. Bassler, Howard A. Stone

**Affiliations:** † Department of Mechanical and Aerospace Engineering, Princeton University, Princeton, New Jersey 08544, United States; ‡ Princeton Institute for the Science and Technology of Materials, Princeton University, Princeton, New Jersey 08544, United States; § Department of Molecular Biology, Princeton University, Princeton, New Jersey 08544, United States; ∥ Howard Hughes Medical Institute, Chevy Chase, Maryland 20815, United States

**Keywords:** diffusiophoresis, biofilms, particle transport, porous media, chemical gradients

## Abstract

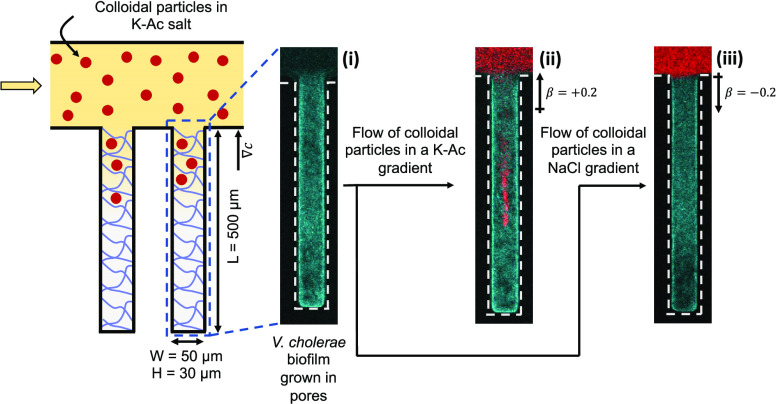

Bacterial biofilms are communities of cells adhered to surfaces. These communities represent a predominant form of bacterial life on Earth. A defining feature of a biofilm is the three-dimensional extracellular polymer matrix that protects resident cells by acting as a mechanical barrier to the penetration of chemicals, such as antimicrobials. Beyond being recalcitrant to antibiotic treatment, biofilms are notoriously difficult to remove from surfaces. A promising, but relatively underexplored, approach to biofilm control is to disrupt the extracellular polymer matrix by enabling penetration of particles to increase the susceptibility of biofilms to antimicrobials. In this work, we investigate externally imposed chemical gradients as a mechanism to transport polystyrene particles into bacterial biofilms. We show that preconditioning the biofilm with a prewash step using deionized (DI) water is essential for altering the biofilm so it takes up the micro- and nanoparticles by the application of a further chemical gradient created by an electrolyte. Using different particles and chemicals, we document the transport behavior that leads to particle motion into the biofilm and its further reversal out of the biofilm. Our results demonstrate the importance of chemical gradients in disrupting the biofilm matrix and regulating particle transport in crowded macromolecular environments, and suggest potential applications of particle transport and delivery in other physiological systems.

## Introduction

Bacterial biofilms are ubiquitous in nature, industry, and medicine. Biofilms can be detrimental to human health; over 80% of bacterial infections are enabled by bacterial biofilms.^1^ Within the human body, biofilms typically form on surfaces, even the narrow spaces between teeth or in crevices in the small intestine.^2^ A defining feature of a biofilm is a three-dimensional extracellular polymer matrix that functions as a barrier to the import of chemicals, such as antimicrobials, making biofilm cells recalcitrant to treatment; consequently, biofilms are difficult to remove from surfaces.^3,4^ Moreover, the chemical, physical, and mechanical properties of a biofilm matrix are heterogeneous, which hinders full understanding and, consequently, development of effective strategies for eradication. The use of micro and nanoparticles for the disruption of biofilms has been widely studied.^5−8^ Nanoparticles offer many advantages due to the ease of synthesizing them with controlled size, shape, material, chemical, and other properties, including opportunities for active drug or ingredient encapsulation. We are not aware, however, of attempts to use chemical gradients to drive particles into biofilms, which is a theme we develop in this paper.

Electrolytes, such as ordinary salts, e.g., NaCl, are present in many solutions, including where biofilms are present. One common role of salt is to screen the charge on surfaces, such as that of the polymers that make up the biofilm matrix. Nevertheless, what is less appreciated is that chemical gradients, both naturally occurring or purposefully created, can induce the motion of suspended colloidal particles. One of several unexplored mechanisms for controlling and enhancing transport of particles in biofilms is through a physicochemical process called diffusiophoresis,^9−12^ which refers to the directed migration of particles (speed, *v*_p_) in a gradient of chemical species. Diffusiophoresis has been reported for polystyrene particles, vesicles, and even bacteria in various electrolytes and over a wide range of geometries.^13−17^

As chemical gradients commonly arise naturally in and around biofilm extracellular matrices,^18^ diffusiophoresis has the potential to transport particles into or out of biofilms. Because of the chemical gradient, there are osmotic effects that contribute a so-called “chemiphoretic” transport mechanism to the particle movement relative to the fluid. Also, for electrolytes, differences in diffusivities between anions and cations generate electrical potentials that enable the movement of a charged particle through an electrophoretic force. The resulting movement is usually described in one dimension in terms of the particle speed *v*_p_ = Γ_p_ d/d*x* ln *c*, where, in electrolyte solutions, *v*_p_ is proportional to the gradient of the logarithm of the concentration field and Γ_p_ is referred to as the diffusiophoretic mobility. For the cases where concentration gradients of a 1:1 electrolyte (i.e., *Z*_–_:Z_+_ = 1:1, where *Z*_–_ and *Z*_+_ are the anion and cation in the electrolyte) drive the particle motion, and with ϵ, μ, *k*_B_, *T*, *e*, and ζ_p_, respectively, the electrical permittivity, fluid viscosity, Boltzmann constant, absolute temperature, elementary charge, and the zeta potential of the (particle) surface, the diffusiophoretic mobility (Γ_p_) is given by
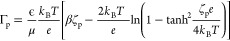
1This equation is based on the assumption that a thin electrical double layer, where counterions are at a higher concentration near a charged surface than they are in the bulk solution, is much smaller than the particle radius.^19^ The motion of particles through the electrophoretic component is dictated in eq 1 by the diffusivity difference factor, β, given by β = (*D*_+_ – *D*_–_)/(*D*_+_ + *D*_–_), where *D*_+_ and *D*_–_ represent the diffusion coefficients of the cation and anion, respectively. The two mechanisms, electrophoresis (the first term in eq 1) and chemiphoresis (the second term in eq 1), together account for the diffusiophoretic transport of a particle in a chemical gradient. We note that the chemiphoretic contribution to diffusiophoresis is always positive, meaning that the particle motion is always toward higher chemical concentrations. Therefore, in the regimes where the electrophoretic contribution dominates over chemiphoresis, the sign of β, which depends on the choice of the salt, can be used to determine the direction of particle motion.

While the majority of studies reporting diffusiophoresis of particles have been performed in low salt or deionized (DI) water conditions, to our knowledge, the transport of particles into biofilms or into a living, crowded macromolecular network is relatively unexplored.^20^ In this work, we investigate externally imposed chemical gradients as a mechanism to transport polystyrene particles into *Vibrio cholerae* biofilms. *V. cholerae* is a globally important pathogen and a notorious biofilm former. We show that preconditioning the biofilm with a prewash step using DI water, which also involves a chemical gradient, is essential for altering the biofilm to take up the micro- and nanoparticles when the prewash is followed by application of a chemical gradient. Using different particles and chemicals, we document particle motion into the biofilm and its further reversal out of the biofilm. Also, our results demonstrate the importance of the chemical species forming the gradient for transport. The results suggest a potential application for the delivery of particles into physiologically relevant and crowded macromolecular biological environments.

## Results and Discussion

### Experimental System

To explore the chemical and physical principles governing the transport of particles into biofilms, we focus on the pathogen *V. cholerae*. There are four crucial *V. cholerae* matrix components, the VPS polysaccharide and the RbmA, RbmC, and Bap1 matrix proteins.^21^ In our experiments, we exploit a commonly used and well-studied *V. cholerae* biofilm-forming strain that carries the *vpvC*^W240R^ mutation as our parent strain. The *vpvC*^W240R^ missense mutation drives hypersecretion of the biofilm matrix, conferring the so-called rugose biofilm phenotype. We also use the *vpvC*^W240R^ Δ*rbmA* double mutant. The RbmA protein links mother and daughter cells together.^22^ Therefore, compared to the densely packed biofilm formed by the *vpvC*^W240R^ strain, the *vpvC*^W240R^ Δ*rbmA* strain produces loosely organized biofilms with increased cell-to-cell distances.^22−24^ Finally, VpsL is required to produce the VPS polysaccharide, and therefore, the *vpvC*^W240R^ Δ*vpsL* double mutant strain is incapable of forming a biofilm. All strains were engineered to constitutively express a bright monomeric fluorescent protein mNeonGreen. The typical LB solution is a solution containing NaCl, yeast extract, and tryptone, with a typical NaCl concentration of ∼100 mM.

**Figure 1:**
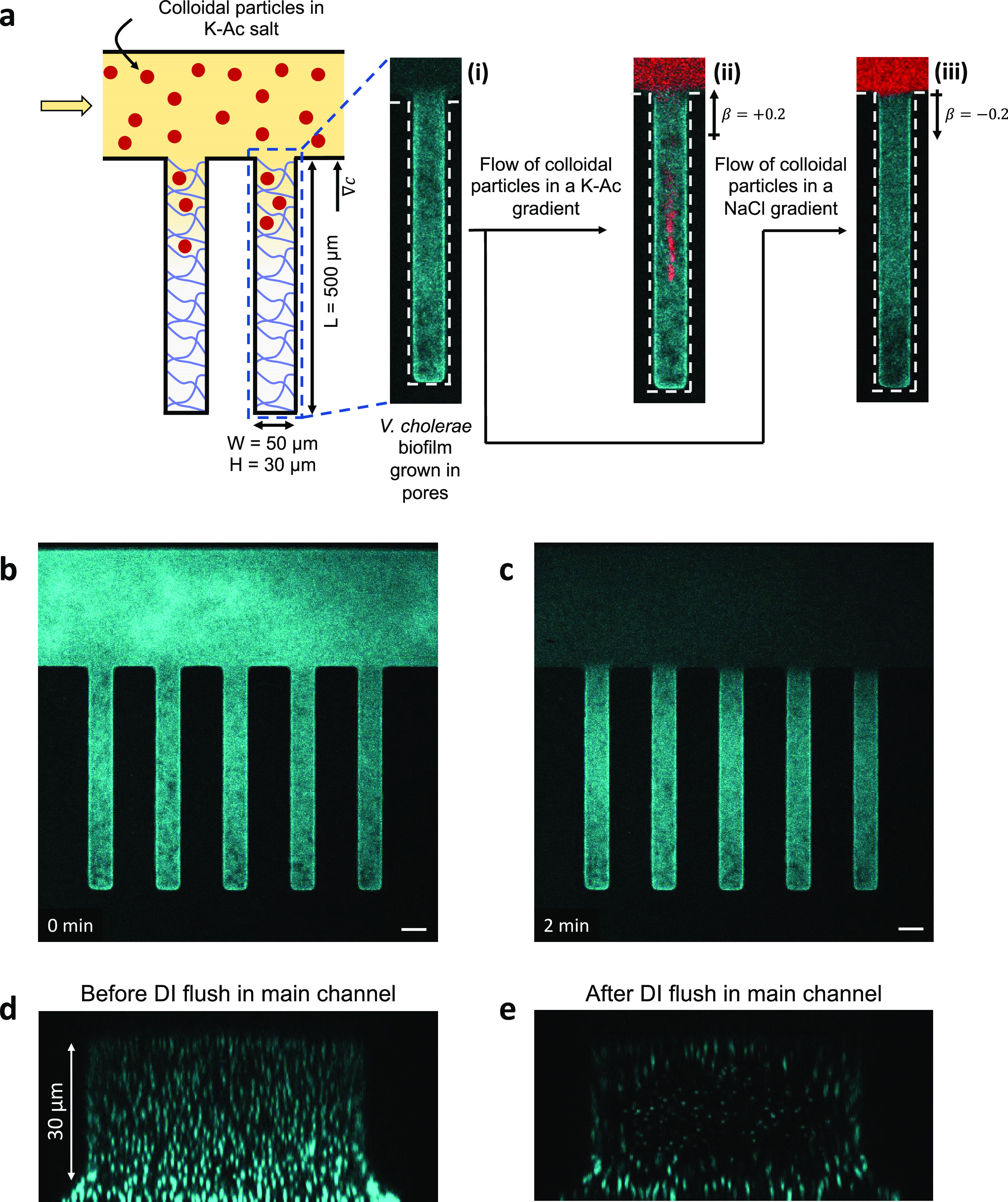
Schematic and images of the experimental setup. (a) Schematic of microfluidic dead-end channel geometry used to test diffusiophoretic particle transport into *V. cholerae* biofilms when there is an imposed chemical gradient. Microparticles are labeled red and bacterial biofilm cells are labeled cyan. Fluorescence images of (i) biofilm-filled dead-end channel, (ii) transport of 100 nm cPS particles into the biofilm-filled dead-end channel in the presence of K-Ac, and (iii) exclusion of particles from the dead-end channel in the presence of NaCl. Fluorescence image of (b) a biofilm-filled dead-end channel before prewashing the main channel with DI water and (c) 2 min after prewashing the main channel with DI water. (d) Channel cross section corresponding to panel (b) of a biofilm-filled dead-end channel before prewashing and (e) cross section corresponding to panel (c) of a biofilm-filled dead-end channel after 2 min of prewashing. Scale bars in panels (b) and (c) equal 50 μm.

We grew *V. cholerae* in microfluidic devices with a dead-end channel geometry (Figure 1a), representative of crevices and cavities. Unless otherwise stated, all experiments were conducted with the *vpvC*^W240R^ Δ*rbmA* strain. The biofilm grows in the dead-end pores, initiating at the edges and growing inward toward the center. Thus, a mechanical strength gradient forms as the biofilm grows from the surface to the center, making the center of the biofilm the weakest region and most prone to rearrangement/loosening. The width and height of the main channel are 250 and 90 μm, respectively. The length, width, and height of the dead-end channels are 500, 50, and 30 μm, respectively. Figure 1a(i) shows a fluorescence image of a dead-end channel that has a *V. cholerae* biofilm (cyan) grown in it; after ∼17 h of growth, the entire microfluidic device is filled with the biofilm, as shown in Figure 1b. We measured the osmotic pressure of a bacterial culture before biofilm formation in the microfluidic channel and found it to be ∼290 mOsm. Additionally, the osmolarity of the *vpvC*^W240R^ Δ*rbmA* biofilm was measured following the method adapted from Szczesny et al.^25^ (details described in the Materials and Methods section), and the obtained value was ∼300 mOsm.

### Chemical Gradient Variations Lead to Distinct Particle Penetration Dynamics in Biofilms

The motivation for the remainder of this work stems from the observation reported in Figure 1a(ii,iii) showing that, following the DI water prewash step (described below) of the main channel, a suspension of FluoSpheres carboxylate-modified polystyrene (cPS) particles (100 nm in diameter, ζ_p_ = −46 mV; marked red) moves into the biofilm-filled dead-end channels from the main channel in the presence of 25 mM potassium acetate (K-Ac) but does not do so in the presence of 25 mM NaCl. The zeta potential of cPS particles was measured using an Anton Paar Litesizer 500^26^ in 25 mM K-Ac solution. Here, we note that the salt and/or solute concentrations of the formed biofilm are unknown, and we make the assumption that the initially provided growth medium is fully consumed. Particularly for NaCl, we assume that most of the salt has been consumed during biofilm formation so that concentration gradients are directed toward the biofilm.

The divergence in behavior of the particles in the presence of the two different salts of the same concentration is consistent with an effect that is a consequence of the difference in diffusivities of the cations and anions of the salts. For K-Ac, the diffusion coefficient of K^+^ is higher than Ac^–^ (*D*_K_ > *D*_Ac_; see Table 1), which, using the understanding of diffusiophoresis, gives a positive β value, resulting in a spontaneous electric field pointing into the dead-end channel (Figure 1a(ii)). In this gradient, negatively charged cPS particles prefer to move away from the high concentration of K-Ac and into the dead-end channels. For NaCl, the diffusion coefficient of Na^+^ is lower than Cl^–^ (*D*_Na_ < *D*_Cl_; Table 1), yielding a negative β value, resulting in the spontaneous electric field directed out of the dead-end channel (Figure 1a(iii)). Thus, our observations are consistent with a diffusiophoretic transport mechanism. Specifically, the choice of electrolyte affects particle motion toward or away from biofilms (see SI for details of diffusiophoretic mobilities set by K-Ac and NaCl).

**Table 1:** Diffusion Coefficients of Ions and the Electrophoretic β Factor Used in the Diffusiophoresis Experiments^11^

ions	diffusion coefficients (10^–9^ m^2^/s)	diffusivity difference factor (β = (*D*_+_ – *D*_–_)/(*D*_+_ + *D*_–_))
K^+^	1.957	K-Ac: +0.285
acetate^–^	1.089	
Na^+^	1.334	NaCl: −0.207
Cl^–^	2.032	

**Figure 2:**
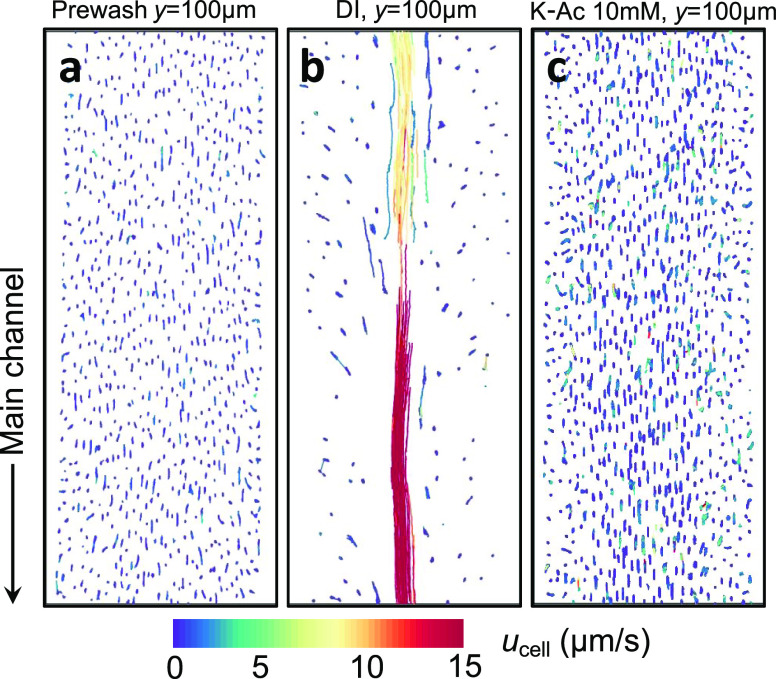
Trajectories of bacterial cells before and after the prewash step. (a) Before the prewash step, (b) after the DI water prewash step, and (c) after the 10 mM K-Ac prewash step. The exposure time for all cell velocimetry is 30 ms and the streaklines represent the displacements during this timeframe. *y* = 100 μm represents the distance into the pore at which this measurement is made. The color bar designates the speed (μm/s) with which bacterial cells move toward the main channel. The width of each plot represents 50 μm.

To establish an externally imposed solute gradient in a uniform and consistent manner across a biofilm, we needed to confine it only to the dead-end channels. To do this, we employed a prewash step in which we flush DI water through the main channel at a flow rate of 0.3 mL/min, corresponding to an average flow speed of 0.2 m/s for 2 min (Figure 1c). The prewash step makes the biofilm more porous, as shown in Figure 1d,e. In particular, Figure 1d shows the cross section of the biofilm across the height of the dead-end channel. Note that the biofilm remains present along the edges of the channel while the center of the channel has larger spacing (porosity) between the bacteria (Figure 1e). Indeed, if the prewash step is performed with either 10 mM K-Ac or 10 mM KCl, no “loosening” occurs, and particles cannot subsequently penetrate (Figures 2 and S1). Conversely, when we prewash the biofilm with a solution containing an uncharged molecule, glucose, particles penetrate the biofilm. After prewashing with glucose, and imposition of the K-Ac gradient, we observe the movement of particles into the biofilm-filled dead-end pores. This result is an indication that charged solutes indeed play a role in making the biofilm resistant to penetration from the external environment.

To confirm the effects of salt on the modification of the biofilm matrix inside the biofilm-filled channels, we performed high-speed confocal imaging to monitor the motion of individual bacterial cells inside the biofilm-filled channels, as shown in Figure 2. We recorded the movements of the cells in the biofilm-filled pores (the scale bar indicates the speed of the cells) before and after the DI water or a 10 mM K-Ac prewash step. Before the prewash, the bacterial cells do not show any significant motion and remain stable inside the biofilm in the dead-end pores (Figure 2a). After the 2-min DI water prewash step at 0.3 mL/min, the cells along the centerline of the biofilm respond to the flush and rapidly move out of the dead-end pores into the main channel (Figure 2b) with speeds about 10 μm/s. In contrast, when we prewash the main channel with a 10 mM K-Ac solution for the same time and at the same flow rate, the cells respond minimally (0–3 μm/s) (Figure 2c). Presumably, during the DI water wash step, ions are stripped away from the biofilm matrix, eliminating its intrinsic salt gradient, which reduces the integrity of the matrix, and allows the bacteria to escape. We note that the strain used in the experiment is locked in the biofilm-forming state and nonmotile. Thus, cell escape cannot be a consequence of motility/chemotaxis. This finding signifies the importance of the prewash step in preconditioning the biofilm to uptake cPS particles through diffusiophoresis. Next, we present systematic experiments changing the applied chemical gradient and the particle size to illustrate the physical processes. Our extensive experiments using various salts and ionic concentrations allowed us to systematically alter and measure the influence of ionic gradients on particle movement into biofilms.

### Twenty Nanometer Particles Move into *V. cholerae* Biofilms in the Presence of K-Ac

**Figure 3:**
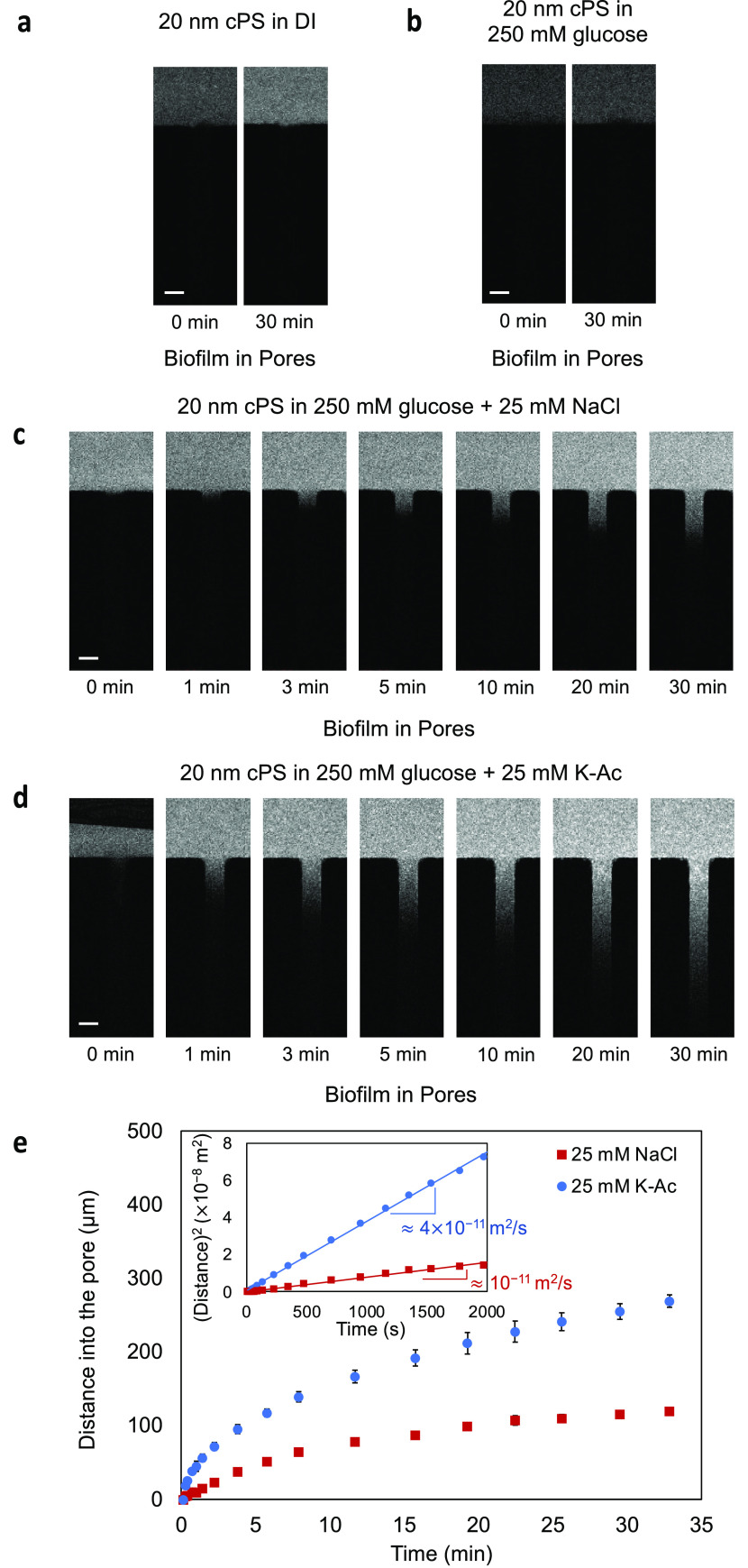
Sequential images of migration of 20 nm cPS particles into biofilm-filled dead-end channels in the presence of (a) DI water, (b) 250 mM glucose, (c) a NaCl gradient, and (d) a K-Ac gradient. (e) Plot of distances moved by 20 nm cPS particles over 30 min in the presence of NaCl and K-Ac gradients. Inset: plot of squared distance versus time. The slopes of the linear graphs represent mobilities (diffusion and diffusiophoresis) of 20 nm cPS particles in the presence of NaCl and K-Ac gradients, as designated. Scale bars in panels (a–d) equal 50 μm.

Since it is known that the *V. cholerae* biofilm extracellular matrix is negatively charged,^27^ we wondered whether the negatively charged particles were being repelled. To test this hypothesis, we monitored the movement of 20 nm cPS particles in the presence of DI water, the uncharged solute glucose, and different concentrations of salt. When the particles are present in DI water or in the uncharged glucose solution, they did not move into the biofilm-filled dead-end channels over 30 min (Figure 3a,b). By increasing the concentration of electrolytes in the solution, the “effective” charges of the particles and that of the extracellular matrix are lowered, through the charge (or Debye) screening effect, which reduces repulsion between the particles and the extracellular matrix. We found that 5 mM NaCl does not provide sufficient charge screening for the particles to move into the biofilm (Figure S2). However, increasing the concentration of NaCl to 25 mM enabled the particles to move into the biofilm-filled dead-end channels (Figure 3c). Over a 30-min time period, particles continuously moved into the biofilm-filled dead-end channels (Figure 3c). Changing the externally imposed solute from NaCl to K-Ac caused the particles to move more deeply into the biofilm over the same time period (Figure 3d). We attribute this increased movement into the biofilm to the choice of externally imposed salt gradient. As explained above, the salt gradient establishes a spontaneous electric field that drives the cPS particles to move in one direction or the other (the chemiphoretic effect always moves particles toward higher solute concentration). In the NaCl gradient, the negatively charged particles should move toward the high concentration of NaCl and away from the dead-end channel. Thus, any particle movement into the dead-end channel in NaCl is purely by diffusion. By contrast, for K-Ac, there is an additional diffusiophoretic effect that drives particles into the dead-end channel.

In order to illustrate the difference between transport processes driven by NaCl and K-Ac gradients, we can assess the diffusiophoretic mobilities of particles in chemical gradients. (Figure S3). For the 20 nm cPS particles, the Stokes–Einstein diffusivity is  m^2^/s, where *a* is the radius of particles (μ = 0.001 Pa·s is used for the liquid viscosity). The diffusivity of particles is comparable to the calculated diffusiophoretic mobility (Γ_p_ ≈ 8 × 10^–11^ m^2^/s; eq 1) set by K-Ac (Figure S3).

We measured and plotted the particle entrainment distance versus time, for both the NaCl and K-Ac experiments (Figure 3e). The particle entrainment distance is calculated by analyzing kymographs of the recorded videos. The trajectory is obtained by thresholding the image and tracking the location of the particle front (i.e., frontmost particle) in and out of the pore. The squared entrainment distance shows a linear trend with time (Figure 3e: inset) for both salts, and the slopes of the linear graphs provide typical scales of the particle mobilities (diffusion or diffusiophoresis). The values of the measured particle diffusivity (≈10^–11^ m^2^/s; in NaCl) and the measured diffusiophoretic mobility (≈4 × 10^–11^ m^2^/s; in K-Ac) are approximately  and , respectively. After the DI water prewash step, the viscosity of the loosened biofilm is expected to be lower than the viscosity of the original biofilm, but higher than that of DI water. From the measured mobilities ( and ) that are half the values of the calculated mobilities, we can suggest that the viscosity of the loosened biofilm is μ ≈ 0.002 Pa·s, which is twice the viscosity of DI water (μ ≈ 0.001 Pa·s). To explore the effect of diffusiophoresis on the biofilm matrix further, we examine particles of larger sizes and in different K-Ac concentration gradients.

### Diffusiophoretic Transport of Particles into Biofilm-Filled Dead-End Channels is Particle Size and Salt Concentration Dependent

**Figure 4:**
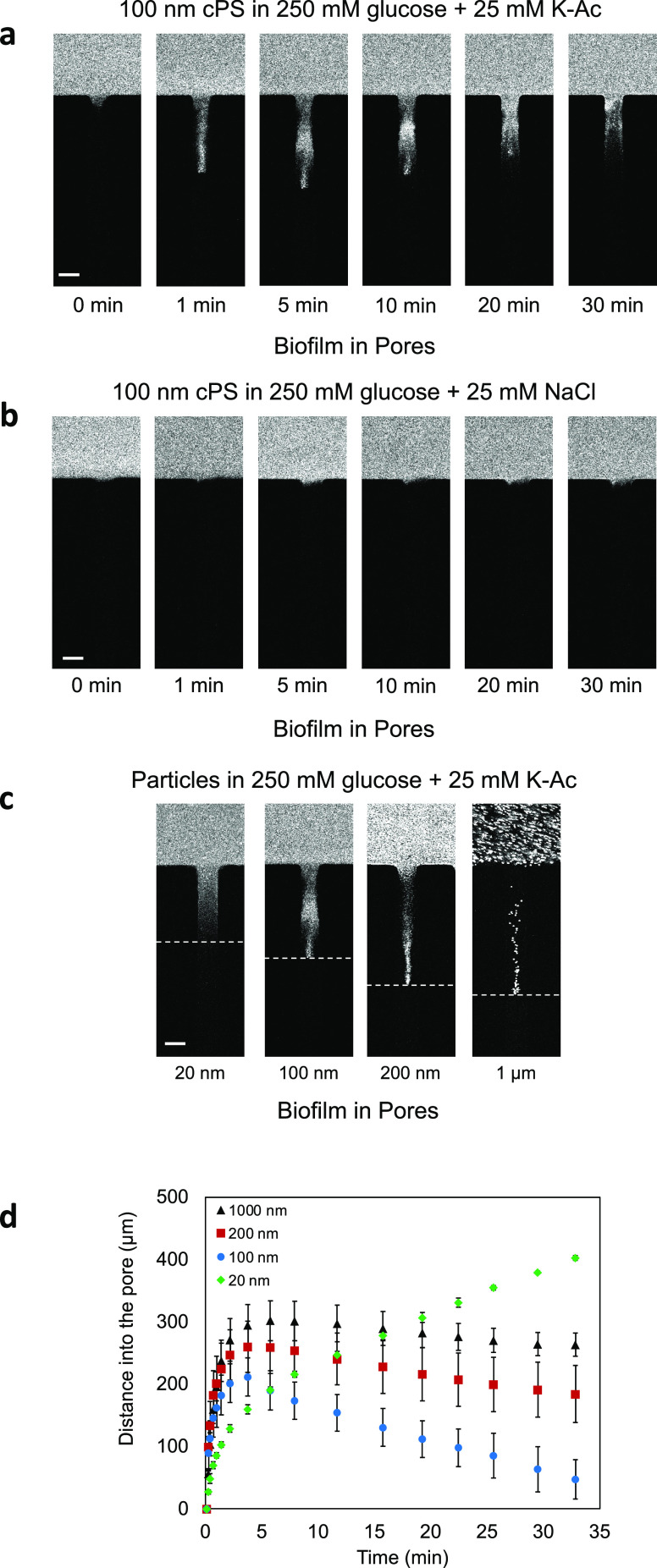
Transport of microparticles into biofilm-filled dead-end channels. Time sequence images of 100 nm diameter cPS particle migration into biofilm-filled dead-end channels in the presence of (a) K-Ac and (b) NaCl gradients. (c) Fluorescence images of carboxylate polystyrene particles of different diameters ranging from 20 nm to 1 μm at *t* = 5 min in the presence of a K-Ac gradient. (d) Plot of distances moved by particles of various sizes over 30 min in the presence of a K-Ac gradient. Scale bars in panels (a–c) equal 50 μm.

Due to the confinement effect^20^ of the biofilm matrix, we expected that larger-diameter particles would be unable to penetrate the biofilm or would penetrate inward to a shorter distance than smaller-diameter particles. However, 100 nm (diameter) cPS particles in gradients of K-Ac penetrated the biofilm (Figure 4a). The particles did not penetrate the entire width of the channel but were localized toward the center as they moved into the biofilm-filled dead-end channels. Moreover, the particles reversed their direction of motion at long times (>5 min) (Figure 4a). When the 100 nm diameter cPS particles were present in a NaCl gradient, no movement into the biofilm-filled dead-end channels occurred over the time course (Figure 4b). This result, by comparison with that in Figure 3c, suggests that the matrix pores are too small for the large particles to move into the biofilm-filled dead-end channel through diffusion. When an external force is provided in the form of a diffusiophoretic K-Ac chemical gradient, the 100 nm particles are able to penetrate the biofilm matrix.

While the movement of particles, vesicles, and bacteria via chemical gradients in free solution has been shown under low salt or DI water conditions,^13−15^ the localization of the particles in a complex polymeric environment and the reversal in their direction of motion in biofilms (Figure 4a) have not been reported to our knowledge. Recently, Doan et al. showed the movement of amine-functionalized polystyrene particles into dead-end channels filled with a collagen matrix.^20^ They showed that as the particle size increases, the mobility of the particles reaches a maximum and then decreases as the matrix boundary confinement prevents the particles from moving in more deeply.^14^ In Figure 4d, in the presence of a K-Ac gradient, we document the movement of 20, 100, 200, and 1000 nm diameter particles in the dead-end channels over 30 mins. To our surprise, as the size of the particle is increased, the particles moved further into the biofilm-filled dead-end channels (Figure 4c). Furthermore, 20 nm cPS did not show reversal behavior while 100 nm diameter and larger particles reversed their transport direction at ∼5 min. Our results are consistent with size-dependent diffusiophoresis that occurs under low salt and DI water conditions.^14^

**Figure 5:**
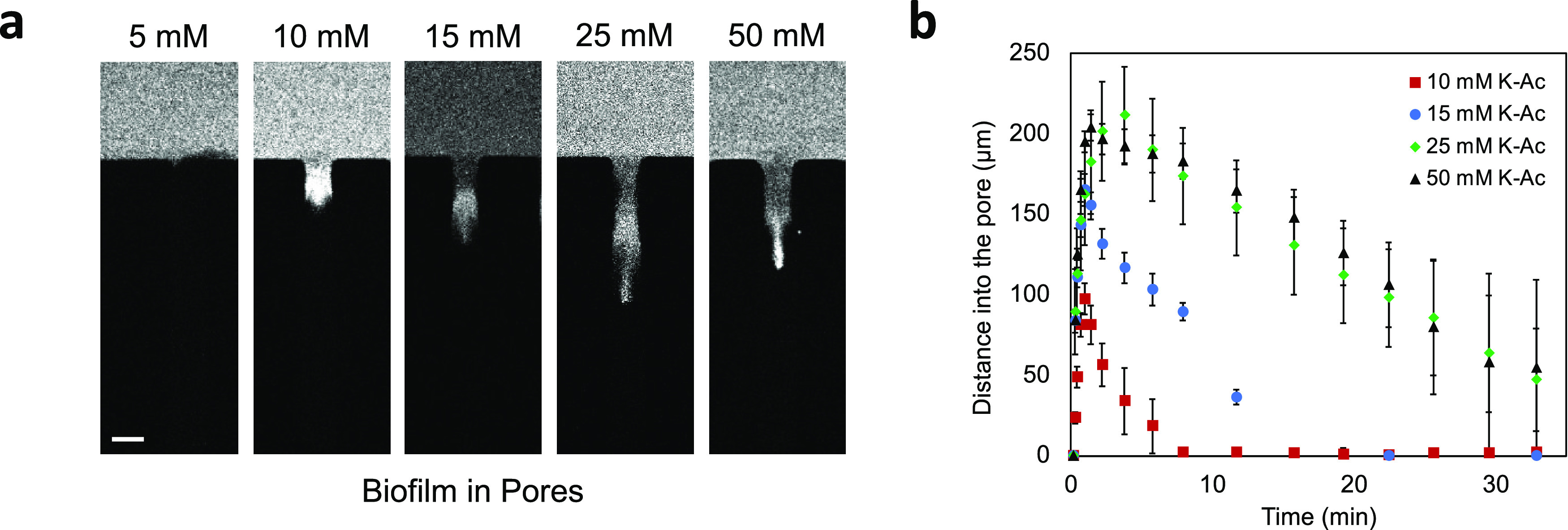
Concentration-dependent transport of particles into biofilm-filled dead-end channels. (a) Fluorescence images of 100 nm diameter cPS particles in different K-Ac concentrations at the inlet (5, 10, 15, 25, and 50 mM). The snapshots are taken at 5 min after the introduction of the gradient. (b) Plot of distances moved by 100 nm diameter cPS particles for different initial K-Ac concentrations. Scale bar in panel (a) equals 50 μm.

To explore the concentration effect of diffusiophoresis in the biofilm matrix, we characterized the movement of 100 nm diameter cPS particles in varying concentration gradients of K-Ac. Our results show that the distance traversed by the particles was proportional to the imposed chemical gradient (Figure 5a,b). Consistent with the control experiment reported in Figure S2, we note that for a low concentration of salt, for example, 5 mM K-Ac, the particles do not penetrate, a result we assume is tied to charge repulsion because of the negative charge of the biofilm matrix (Figure 5a). When the salt concentration is increased sufficiently to screen the charges on the polymer matrix, the particles migrate into the biofilm in accordance with diffusiophoresis.

### Comparison of Particle Movement into Biofilms Formed by the *V. choleraevpvC*^W240R^ and *vpvC*^W240R^ Δ*rbmA* Strains

**Figure 6:**
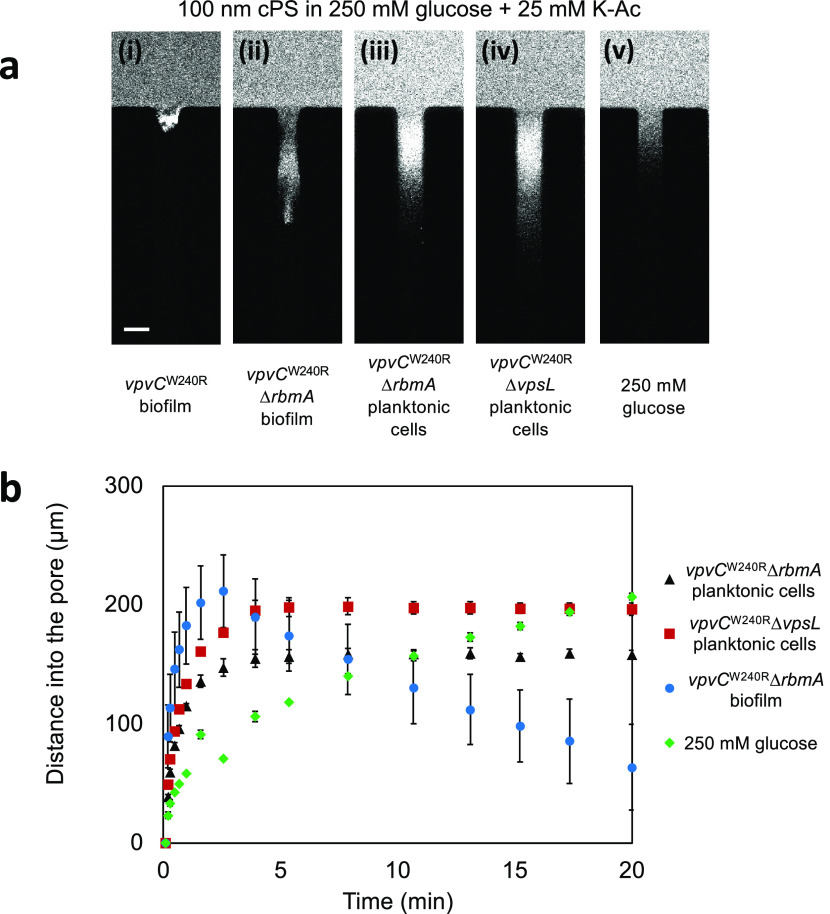
Fluorescence images of 100 nm diameter cPS particles in the presence of 250 mM glucose and 25 mM K-Ac and the effects on biofilms formed by different *V. cholerae* strains. The strains and conditions in panel (a) are (i) *vpvC*^W240R^ biofilm, (ii) *vpvC*^W240R^ Δ*rbmA* biofilm, (iii) *vpvC*^W240R^ Δ*rbmA* planktonic cells, (iv) *vpvC*^W240R^ Δ*vpsL* planktonic cells, and (v) only 250 mM glucose. (b) Plot of distances traveled by 100 nm diameter cPS particles over 20 min into the dead-end channels containing the designated *V. cholerae* strains or glucose. Scale bar in panel (a) equals 50 μm.

To confirm that the behavior of particles we revealed is indeed due to the presence of the biofilm matrix coupled with the imposed chemical gradient, we exploited *V. cholerae* mutants with different biofilm matrix properties. Regarding the *vpvC*^W240R^*V. cholerae* strain that makes a robust biofilm, 100 nm diameter cPS particles did not penetrate into the biofilm-filled dead-end channels (Figure 6a(i)). This result suggests that in addition to the externally imposed gradients and DI water prewash step, the composition of the biofilm matrix dictates the penetration of particles. In the absence of the RbmA protein in biofilms formed by the *vpvC*^W240R^ Δ*rbmA* mutant (Figure 6a(ii)), penetration of particles into the biofilm occurred.

Next, we filled the dead-end channels with a planktonic (i.e., nonbiofilm) culture of the *vpvC*^W240R^ Δ*rbmA* mutant (Figure 6a(iii)). In this case, particles penetrated through the entire width of the channel. When we filled the dead-end channel with a planktonic culture of the *vpvC*^W240R^ Δ*vpsL* mutant, which is incapable of making a matrix, particles were able to traverse even further into the dead-end channel (Figure 6a(iv)). These results show that the biofilm matrix plays a role in resisting the penetration of particles. Our control experiment in which bacteria were absent shows movement of particles akin to normal diffusiophoresis as reported in several previous studies (Figure 6a(v)).^12−14^ Moreover, there is no localization at the center of the channel and there is no reversal behavior at long times. These results show that the presence of the biofilm is essential for the localization during particle penetration and for particle reversal behavior, which demonstrates a unique transport behavior in a crowded macromolecular environment.

### Note on Biofilm Particle Penetration Dynamics

For a given length of dead-end channel, we can estimate the time required for the K-Ac gradient to dissipate. Taking the length of the channel to be 500 μm and the diffusion coefficient of K-Ac salt to be *D* ∼ 10^–9^ m^2^/s, we estimate the time for the gradient to dissipate as (distance)^2^/*D* ∼ 4 min. This timescale is of the same order of magnitude as the time during which particles reverse their directions of motion, i.e., in the first 5 min, when the gradient is initially imposed, the cPS particles respond to the gradient and move into the biofilm-filled channels. At long times (>5 min), the K-Ac gradient is dissipated, the particles are no longer able to penetrate the biofilm matrix, and thus they reverse their directions of motion and move out of the dead-end channel. The interplay between the diffusiophoretic force and the interaction of particles with the biofilm extracellular matrix is hypothesized to lead to this reversal in motion of the particles. At short times, the diffusiophoretic force dominates and pushes particles into the biofilms, whereas at long times (>5 min), the steric features of the biofilm, combined with the chemical gradients established by K-Ac treatment, push particles out of the biofilm. We take the particle transport behaviors of 20 and 100 nm particles as our examples: In Figures 3c and 4b, in the presence of an unfavorable NaCl gradient, 20 nm particles move into the biofilm matrix through diffusion. However, 100 nm particles are unable to move into the matrix via diffusion because they are larger than the biofilm matrix pores. As diffusion is force-free, the size of the biofilm pores determines whether or not particles are transported into the biofilm. In the case in which a K-Ac gradient is imposed, the diffusiophoretic force enables the movement of both the 20 and 100 nm into the biofilm matrix. Reversal in the direction of motion occurs only for the 100 nm particles. After the dissipation of the diffusiophoretic gradient, and hence the diffusiophoretic force, the steric features of the biofilm eject the 100 nm particles giving rise to the reversal behavior that is only observed for the larger particle sizes.

In addition to the imposed chemical gradient, we highlight the prewash step by DI water (Figure 1), which was used to confine the biofilms to the dead-end channels. We hypothesize that the absence of ions in DI water generates a gradient of ions flowing from inside the biofilm out into the main channel. In creating this gradient, bacteria inside the biofilm-filled dead-end channels demonstrate a fast flow out of the dead-end channel into the main channel during the prewash step (Figure 2). The DI water in the main channel acts as a sink to strip away the ions or solutes that are crucial to maintain the biofilm integrity, increasing biofilm porosity and allowing resident bacteria to escape. Moreover, this treatment also makes the biofilm susceptible to penetration from the cPS particles supplied after the prewash step.

To confirm that it is indeed the absence of ions that leads to penetration and subsequent reversal of particle transport, we performed experiments in which we used 10 mM K-Ac in the prewash step (Figure S1). Particles did not penetrate into the biofilm and there was no alteration of the bacteria in the biofilm in response to the prewash. These findings are key as they show that the choice of solution used in the prewash step is critical for subsequent particle penetration into the biofilm and reversal of the direction of motion of the particles. Lastly, this result also eliminates any possible effects of shear being responsible for particle transport and/or affecting the biofilm integrity.

## Conclusions

We have shown that particles can be transported into *V. cholerae* biofilms by exploiting chemical gradients via a diffusiophoretic mechanism. A DI water prewash step effectively “loosens” the biofilm and makes it permeable to the uptake of micro- and nanoparticles that are delivered in the context of a favorable chemical gradient. We also show that the intrinsic nature of the biofilm matrix is responsible for the uptake or blocking of microparticles. Particles penetrate biofilms prepared with the *vpvC*^W240R^ Δ*rbmA* mutant; however, they do not enter *vpvC*^W240R^ biofilms irrespective of the prewash step or favorable chemical gradient. Finally, our results signify the importance of chemical species in regulating particle transport in crowded macromolecular environments and suggest a potential application in delivery into harmful bacterial biofilms, which we believe represents a valuable new research opportunity.

## Materials and Methods

### Strains and Growth Conditions

All *V. cholerae* strains used in this study are derivatives of the wild-type *V. cholerae* O1 biovar El Tor strain C6706, harboring a missense mutation in the *vpvC* gene (*vpvC*^W240R^) that elevates c-di-GMP levels, conferring the so-called rugose biofilm phenotype. The gene encoding the monomeric fluorescent protein mNeonGreen was constitutively expressed under the P*tac* promoter along with the spectinomycin resistance gene inserted at the neutral genome locus *vc*1807. All strains were grown in Luria-Bertani (LB) broth (Lennox) at 37 °C with shaking.

### Materials

20 nm, 100 nm, 200 nm, and 1 μm diameter FluoSpheres carboxylate-modified polystyrene microspheres (cPS), red (ex/em: 580/605) were purchased from Thermo Fisher Scientific. NaCl, glucose, and LB (Lennox) broth were purchased from Sigma-Aldrich. K-Ac was purchased from MP Biomedicals. Poly(dimethylsiloxane) (PDMS) was purchased from Dow Corning (Sylgard 184).

### Microfluidic Experimental Protocol

The PDMS dead-end microchannels were prepared using standard soft lithography techniques. Briefly, a silicone elastomer base and an elastomer curing agent were mixed in a 10:1 ratio. The degassed mixture was poured onto the silicon wafer molds and allowed to cure for a minimum of 3 h. The main microchannel has a dimension of 250 μm × 90 μm (*W* × *H*). The dead-end channels have dimensions of 500 μm × 50 μm × 30 μm (*l* × *w* × *h*). Fluorescence images were recorded every 2.5 s for 30 min using a confocal laser scanning microscope (Leica TCS SP5).

### Biofilm Growth in Microchannel Dead-End Channels

*V. cholerae* strains were grown overnight in 5 mL of LB broth at 37 °C with shaking. Microchannels were filled with the bacterial culture from the inlet. After the main channel was filled, the exit was blocked, and pressure was applied at the inlet to force the flow of the bacterial culture into the dead-end channel. After filling, the bacteria were given 1 h to attach to the surface of the microchannel. Sterile LB was injected into the channel at a flow rate of 30 μL/h using a syringe pump. The flow was maintained overnight (∼17 h) to allow biofilm development. Subsequently, DI water at 0.3 mL/min was injected into the microfluidic device for 2 min to detach biofilms from the main channel of the device, leaving only the dead-end channels filled with biofilm. We chose 2 min as the preferred time for the prewash step as the majority of the biofilm was removed from the main channel within that time. When nonbiofilm cells were required, bacteria from overnight cultures were introduced into the dead-end channels and the particle suspension was immediately supplied.

### Flow of Particles in Dead-End Channels

The FluoSpheres carboxylate-modified microspheres were diluted 100-fold before use. Briefly, 990 μL of the required solute solution (DI water, glucose, or salt solution) was added to 10 μL of microparticle suspension in a 1.5 mL Eppendorf tube. Following the main channel prewashing step, a bubble was introduced in the microchannel before the introduction of the microparticle suspension at a flow rate of 30 μL/h. The introduction of the bubble enabled a constant solute concentration to be established while performing the experiment.^11^

### Osmolarity Measurements for Bacterial Suspensions and Biofilms

Osmotic pressure measurements were performed using a Precision Systems Micro Osmometer (5004 μ-Osmette). The *vpvC*^W240R^ Δ*rbmA* strain was grown overnight in 5 mL of LB broth at 37 °C with shaking. For the bacterial suspension osmolarity measurement, 50 μL of the overnight grown culture was used directly. For the biofilm osmolarity measurement, we followed steps adapted from Szczesny et al.^25^ Instead of the continuous-flow microfermentors used,^25^ we grew the biofilm using a centimeter-scale flow channel. The suspension was grown overnight and flowed into, and completely filled, a 3.5 cm (width) × 5 cm (length) × 2 mm (height) flow channel. The channel was made by bonding a PDMS block with a 2-mm PDMS spacer on a 50 mm × 75 mm slide glass. After waiting 1 h to allow the bacterial cells to attach to the surfaces of the channel, LB solution was connected to the channel and flowed into it at a volumetric flow rate of 2 mL/h. After 17 h, the LB flow was stopped, and the remaining LB in the channel was slowly removed by the back pressure of the syringe. Subsequently, the PDMS block was gently detached, and the biofilm biomass was recovered from both the PDMS and the glass surfaces using a cell scraper. The biofilm sample was subjected to centrifugation at 2700*g* for 15 min, and the supernatant (50 μL) was collected for the osmolarity measurements.

## Supporting Information Available

Sequential images of 100 nm cPS in the presence of 250 mM glucose and 25 mM K-Ac (Figure S1), images showing that 20 nm cPS particles in the presence of 290 mM glucose and 5 mM NaCl are unable to penetrate biofilm-filled dead-end pores (Figure S2), diffusiophoretic mobilities plotted versus surface zeta potentials (Figure S3), and experimental details (PDF)
