# Supplementary material for: Diffusiophoretic Particle Penetration into Bacterial Biofilms
Source: ACS Appl Mater Interfaces. Author manuscript; Available in PMC 2023 Aug 9. (PMC10360038; doi:10.1021/acsami.3c03190)
Supplement: Supplemental [file NIHMS1919660-supplement-Supplemental.pdf]

Supplementary Information

Diffusiophoretic Particle Penetration into Bacterial Biofilms

Ambika Somasundar[1,2], Boyang Qin[1,3], Suin Shim[1], Bonnie L. Bassler[3,4] and Howard A. Stone[1]*

[1]Department of Mechanical and Aerospace Engineering, Princeton University, Princeton, New Jersey 08544, United States

[2]Princeton Institute for the Science and Technology of Materials, Princeton University, Princeton, New Jersey 08544, United States

[3]Department of Molecular Biology, Princeton University, Princeton, New Jersey 08544, United States

[4]Howard Hughes Medical Institute, Chevy Chase, Maryland, 20815, United States

*Corresponding author email: hastone@princeton.edu

Pre-washing with a salt solution hinders diffusiophoretic transport of cPS particles

To confirm that it is indeed the absence of ions that leads to penetration and subsequent reversal of particle transport, we perform experiments in which we used 10 mM K-Ac in the pre-wash step. In this experiment, the main channel was flushed with a 10 mM solution of KCl or 10 mM K-Ac (instead of DI water) during the pre-wash step. After the pre-wash step, the main channel was injected with a suspension of 100 nm negatively charged FluoSpheres™ carboxylate-modified (cPS) particles in 250 mM glucose and 25 mM K-Ac. No movement of the particles into the biofilm occurred despite the presence of the diffusiophoretic gradient (Figure S1).

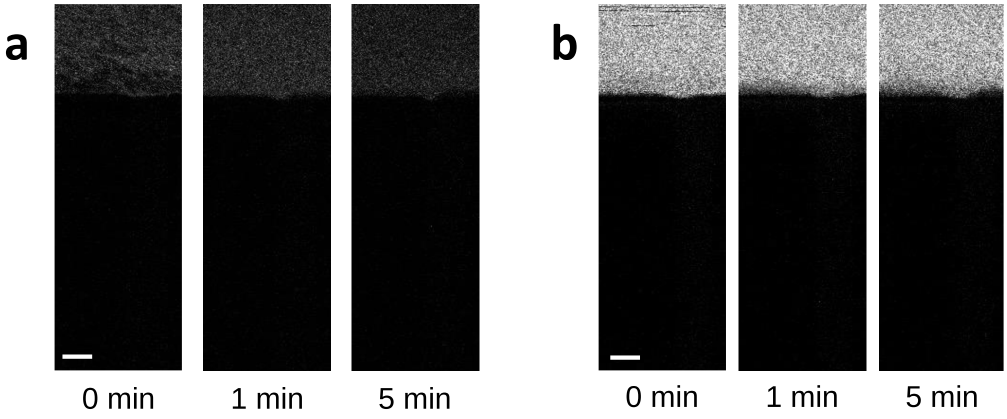

Figure S1. Sequential images of 100 nm cPS in the presence of 250 mM glucose and 25 mM K-Ac show that the particles were unable to move into biofilm-filled dead-end pores. The pre-wash step was performed using (a) 10 mM KCl and (b) 10 mM K-Ac. Scale bar represents 50 µm.

5 mM NaCl does not allow 20 nm cPS particles to penetrate biofilm-filled pores

To test whether a polymer screening effect prevents the biofilm from allowing particles to penetrate it, we injected 20 nm cPS particles in the presence of low concentration of NaCl (5 mM) into the biofilm-filled pores and measured their movement. No penetration into the biofilm occurred over 30 min.

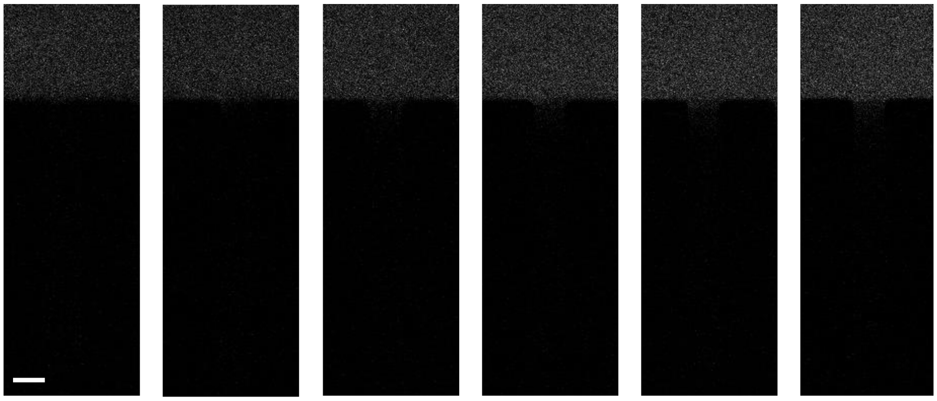

Figure S2. Sequential images show that 20 nm cPS particles in the presence of 290 mM glucose and 5 mM NaCl are unable to penetrate biofilm-filled dead-end pores over 30 min. The pre-wash step was performed using DI water. Scale bar represents 50 µm.

Diffusiophoretic mobilities are established by the NaCl and K-Ac concentration gradients

Diffusiophoretic mobilities of cPS particles in the presence of NaCl and K-Ac concentration gradients can be obtained by solving[1]

$$\Gamma_p = \frac{\epsilon}{\mu}\frac{k_B T}{e}\left[\beta\zeta_p - \frac{2k_B T}{e}\ln\left(1-\tanh^2\frac{\zeta_p e}{4k_B T}\right)\right],$$

where ϵ, μ, k_B, T, e and ζ_p are, respectively, the electrical permittivity, fluid viscosity, Boltzmann constant, absolute temperature, elementary charge, and the zeta potential of the (particle) surface. The sign and magnitude of the diffusivity difference factor $\beta = \frac{D_+ - D_-}{D_+ + D_-}$ dictates the electrophoresis mechanism in diffusiophoresis. The chemiphoretic contribution is always positive, meaning that the direction of motion generated by chemiphoresis is always toward the high concentration of ions. Therefore, within the regimes where electrophoresis makes the dominant contribution, the direction and speed of the particle motion depend on the signs of β and ζ_p. We plot the values of Γ_p versus particle zeta potential in Figure S3, for the cases with NaCl and K-Ac.

The environment created by the "loosened" biofilm in the dead-end pores is more complicated than that of simple 1:1 electrolyte solutions. Nevertheless, we can compare the orders of magnitude of experimental measurements to the values we obtained from simple calculations, as the major observations are diffusiophoretic particle penetration induced by the K-Ac concentration gradient.

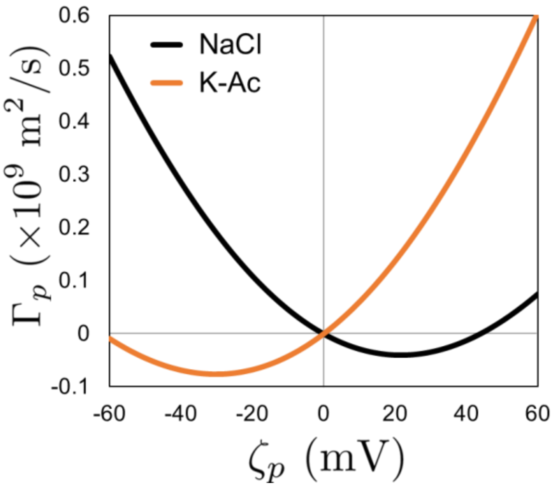

Figure S3. Diffusiophoretic mobilities plotted versus surface zeta potentials. For negative zeta potentials (ζ_p > –60 mV), the mobility established by the K-Ac gradient is negative, meaning that the cPS particles move toward the low concentration region.

References

(1) Prieve, D. C.; Anderson, J. L.; Ebel, J. P.; Lowell, M. E. Motion of a Particle Generated by Chemical Gradients. Part 2. Electrolytes. *J. Fluid Mech.* **1984**, *148*, 247–269.
